# A non-neurodegenerative REM parasomnia with immersive dreaming and dream-reality confusion: a case report

**DOI:** 10.3389/frsle.2025.1659300

**Published:** 2025-09-11

**Authors:** Nazanin Biabani, Martina Mulas, Carlotta Mutti, Sean Higgins, Katarina Ilic, Joshua Benson, Ana Santic, Valentina Gnoni, Panagis Drakatos, Andrea Romigi, Alexander D. Nesbitt, Sharon L. Naismith, Carlos H. Schenck, David O'Regan, Monica Puligheddu, Ivana Rosenzweig

**Affiliations:** ^1^Sleep and Brain Plasticity Centre, Department of Neuroimaging, Institute of Psychiatry, Psychology and Neuroscience (IoPPN), King's College London, London, United Kingdom; ^2^Sleep Disorder Centre, Department of Medical Sciences and Public Health, University of Cagliari, Cagliari, Italy; ^3^Sleep Disorders Centre, Department of Medicine and Surgery, Parma University Hospital and Mario Giovanni Terzano Interdepartmental Centre for Sleep Medicine, University of Parma, Parma, Italy; ^4^Sleep Disorders Centre, Guy's and St Thomas' NHS Foundation Trust, London, United Kingdom; ^5^BRAIN Centre, Institute of Psychiatry Psychology and Neuroscience, King's College London, London, United Kingdom; ^6^Department for Sleep Disorders, Psychiatric Clinic Vrapce, Zagreb, Croatia; ^7^Department of Clinical Research Center for Neurodegenerative Diseases and the Aging Brain, University of Bari ‘Aldo Moro' Pia Fondazione “Card. G. Panico” Tricase (LE), Tricase, Italy; ^8^School of Basic and Medical Biosciences, Faculty of Life Science and Medicine, King's College London, London, United Kingdom; ^9^IRCCS Neuromed Istituto Neurologico Mediterraneo Sleep Medicine Center Pozzilli IS and International Medical University UNICAMILLUS, Rome, Italy; ^10^Department of Neurology, Guy's and St Thomas' NHS Foundation Trust, London, United Kingdom; ^11^Healthy Brain Ageing Program, The Brain and Mind Centre, University of Sydney, Sydney, NSW, Australia; ^12^Minnesota Regional Sleep Disorders Center, and Departments of Psychiatry, Hennepin County Medical Centre and University of Minnesota Medical School, Minneapolis, MN, United States

**Keywords:** REM sleep behavior disorder, dream-reality confusion, immersive dreaming, parasomnia, REM sleep without atonia, melanin-concentrating hormone (MCH), memory consolidation, sleep cognition

## Abstract

**Background:**

REM sleep behavior disorder (RBD) is characterized by loss of normal muscle atonia during REM sleep, often associated with dream enactment behaviors, and is typically a prodromal neurodegenerative condition in middle-aged and older adults. However, emerging case reports and case series suggest that not all RBD presentations follow this trajectory, particularly in younger individuals.

**Case presentation:**

A case of 7-year history of vivid, immersive dreaming perceived as continuous with waking life, accompanied by persistent dream-reality confusion, is described. The patient frequently engaged in reality-testing behaviors and reported significant cognitive fatigue. Video-polysomnography confirmed REM sleep without atonia and a concordant dream re-enactment episode. Neuropsychiatric evaluation ruled out dissociative or psychotic disorders, and no evidence of neurodegenerative disease was observed.

**Conclusion:**

This presented case illustrates a potentially distinct, non-neurodegenerative REM parasomnia phenotype that underscores the need to expand current parasomnia classifications to better capture the diverse cognitive and metacognitive dimensions of REM sleep disorders. Moreover, potential mechanisms underlying the main features of this case, including immersive dreaming and persistent dream-reality confusion, are discussed in relation to hypothesized dysfunction in melanin-concentrating hormone (MCH) signaling.

## 1 Introduction

REM sleep is a discrete neurophysiological state defined by dyssynchronous cortical activation, not that dissimilar to wakefulness, and with muscular atonia (McCarley, 2007). A substantial body of work supports REM sleep's role in procedural memory consolidation, emotional regulation, higher cognition and generative mentation, commonly experienced as dreaming (Stickgold and Walker, 2013). In patients with REM sleep behavior disorder (RBD), patients can be observed re-enacting their dreams due to loss of muscular atonia (due to presumed underlying pathological process in the brainstem region), which otherwise defines this state (Schenck and Mahowald, 2002). Patients with RBD are known to be at significantly increased risk of developing alpha-synucleinopathies, including Parkinson's disease and Dementia with Lewy bodies (Iranzo et al., 2006). Moreover, idiopathic RBD (iRBD) is increasingly viewed as a prodromal, or an early stage, of such disorders, particularly in middle-aged and older adults (Postuma et al., 2009).

However, not all patients with RBD-like symptoms follow this trajectory. It has been noted that a smaller subgroup, often younger, and without neurodegenerative signs, can present with REM sleep without atonia (RSWA), dream enactment, and vivid dreaming, and yet they can remain clinically and cognitively stable over years (Stores, 2008). A more recent case series involving children, adolescents, and young adults found no evidence of neurodegeneration, but did identify a diverse range of comorbidities, including neurodevelopmental disorders (Shukla et al., 2019). Historical pediatric reports remind us that REM-sleep motor phenomena can present outside prodromal neurodegeneration. In particular, early case series described REM-sleep motor disorder in children, underscoring that RBD-spectrum features may occur in younger patients without progressive neurological disease (Sheldon and Jacobsen, 1998). Within this context, our case adds a cognitive-metacognitive phenotype (epic dreaming with dream–reality confusion) to the non-neurodegenerative end of the spectrum. The nosological position of these individuals remains uncertain. Their presentation invites important questions about the breadth of the RBD spectrum and the specificity of its association with synucleinopathy across the lifespan.

Historically, aside from RBD, other dream-related disorders have similarly captured the interest of sleep researchers. For instance, one pathology that is yet to be officially included into any official disorder classification includes reports of abnormal persistence of dream content into waking life (Rassin et al., 2001). In such cases patients have been known to describe dreams of such clarity and realism that they become phenomenologically indistinguishable from lived experience (Mazzoni and Loftus, 1996). This phenomenon, termed dream-reality confusion, has been reported as a feature of several parasomnias (Tuisku, 2020; Gnoni et al., 2020). It has been also reported in narcolepsy-spectrum syndromes, and, more controversially, in dissociative and psychotic conditions (Wamsley et al., 2014). In its most disabling form, dream-reality confusion compels individuals to engage in elaborate reality-testing rituals, thus, undermining their confidence in memory and agency, with profound effects on the quality of their life (Rassin et al., 2001).

The concept of “epic dreaming” similarly remains underexplored. It refers to immersive, continuous dream narratives that, while emotionally neutral and mundane, are subjectively experienced as mentally and physically exhausting (Lin, 2023; Yeh and Schenck, 2000; Schenck and Mahowald, 1995). Unlike nightmares or lucid dreams, epic dreams have been described by affected individuals as notable for their content's ordinariness and their sustained narrative structure (Lin, 2023). Patients often report awakening unrefreshed, with a sense that the night has passed in unrelenting mental activity. Taken together, where these cognitive-affective symptoms intersect with RSWA and dream re-enactments, they may be taken to suggest a broader and more heterogeneous REM parasomnia phenotype than currently represented in the International Classification of Sleep Disorders (ICSD-3; Högl et al., 2018; Sateia, 2014). At present, parasomnia classification is anchored predominantly in behavioral and motor phenomena, with less consideration given to the cognitive and metacognitive correlates of REM disruption (Mahowald and Schenck, 2005).

The present study reports a case with persistent dream reality re-enactment, immersive epic dreaming, and polysomnographically confirmed RSWA with dream re-enactment. The case is of particular interest due to the temporal consistency of the symptoms, the absence of psychiatric comorbidity, and the phenomenological richness of the patient's account. Importantly, RSWA and dream enactment were captured in a polysomnographic study conducted prior to pharmacological intervention. The patient's dreams were reported as being commonly devoid of emotional salience but retained with such fidelity that they repeatedly disrupted post-sleep functioning (see Appendix for some examples). Reality-monitoring deficits were particularly prominent and intrusive.

In exploring this presentation, we draw attention to emerging, albeit theoretical, work on REM-associated memory suppression. Animal studies have proposed that melanin-concentrating hormone (MCH) neurons, which are active during REM sleep, may play a role in attenuating the encoding of REM sleep mentation, possibly by modulating hippocampal plasticity (Izawa et al., 2019). If such mechanisms are indeed operative in humans, dysfunction within this system may permit inappropriate consolidation of dream content, contributing to intrusive post-REM recall, and dream re-enactment (Torterolo et al., 2011). These considerations, whilst speculative, however, advance frameworks that may be valuable in guiding future empirical investigation.

## 2 Case presentation

A right-handed woman in her late 30s presented with a 7-year history of frequent, immersive dreaming experiences indistinguishable from waking reality. The dreams, which occurred four to six times per week, were typically mundane, non-stereotyped, and centered on routine professional or domestic tasks (see Table 1; Supplement for Supplementary Table S1 and Dream Vignette). Notably, they were experienced as continuous with wakefulness. For example, she reported significant post-awakening confusion, often unable to determine whether specific events or conversations had occurred during sleep or wakefulness. Functional impact was marked, the patient routinely consulted digital records (e.g., text messages, emails) to verify whether actions or communications had actually taken place.

**Table 1:** Phenomenological dimensions of immersive dreaming and dream-reality confusion in the present case.

**Phenomenon**	**Patient example**	**Clinical impact**
**Temporal distortion**	Complete workday sequence (commute, work activities, return home) experienced within single REM period	Reports sleep as “second shift;” morning fatigue despite adequate sleep duration
**Narrative coherence**	Logical progression through multiple locations with appropriate social interactions and contextual awareness	Dreams mirror autobiographical memory structure; enhances post-wake believability
**Procedural memory**	Detailed recall of administering medication and pet care with specific tactile sensations	Checked medicine cabinet and pet supplies to verify non-occurrence
**Meta-cognitive confusion**	Dreamed about forgetting to record dreams, creating uncertainty about documentation	Repeatedly reviewed journal entries to establish reality timeline
**Nested false memories**	Multi-layer dream: event → discussing event → following up on discussion	Sent clarifying text to colleague about conversation that never happened
**Professional interference**	Dreamed of work obligations involving superior's belongings with morning deadline	Arrived early to prepare for non-existent task; required explanation to colleagues
**Social confabulation**	Participation in community event with assigned role and identification badge	Uncertainty about attending follow-up meeting that did not exist
**Spatial navigation**	Became lost while navigating waterways but used landmarks to reorient within dream	Engages hippocampal mapping systems; creates compelling sense of genuine experience

This table summarizes key experiential phenomena reported by the patient, with illustrative examples and associated clinical consequences. The domains include temporal distortion, narrative coherence, procedural memory errors, metacognitive confusion, nested false memories, professional interference, social confabulation, and spatial navigation. Each reflects a distinct aspect of the patient's immersive dreaming and dream-reality confusion, highlighting the functional and cognitive impact of persistent REM mentation on waking life. These examples underscore the metacognitive disruptions and behavioral consequences of non-pathological, yet disabling REM parasomnia.

The patient denied any history of trauma, neurologic or psychiatric disorders, or substance use. There was no reported history or clinical suspicion of a prior sleep disorder. Her developmental history included mildly delayed ambulation and persistent childhood tactile hypersensitivity, with no formal diagnosis. Medical history was notable for polycystic ovary syndrome and gastrointestinal dysmotility, treated with prucalopride. She was not taking any psychotropic medications at the time of referral. Cognitive screening with the Addenbrooke's Cognitive Examination (ACE) yielded a perfect score of 100/100, indicating intact global cognitive functioning.

An overnight video-polysomnography (vPSG) was performed prior to pharmacological intervention. Total sleep time was 409 min with preserved sleep architecture. Sleep efficiency was high at 96.3%, with 14.5 min of wake after sleep onset (WASO), The Epworth Sleepiness Scale score of 12/24, arousal index of 14.7 events/hour, apnea–hypopnea index (AHI) of 1.3 events/hour, and periodic limb movement index (PLMI) of 5.1 events/hour were recorded. Stage N1 sleep comprising 12.5% of total sleep time (TST), N2 48.4%, and N3 20.0% were reported. REM sleep accounted for 22.3% of total sleep time. REM latency was 98 min, and sleep latency was 4.5 min. A 15-day actigraphy assessment preceding the vPSG showed a stable and regular sleep–wake pattern, with an average time in bed of 9 h and 11 min, and average sleep duration of 7 h and 44 min, with infrequent and brief napping. A multiple sleep latency test (MSLT) demonstrated sleep onset in all four nap opportunities, with a mean sleep latency of 9.7 min and no sleep-onset REM periods (SOREMPs). Notably, tonic and phasic electromyographic (EMG) activity during REM epochs was observed, consistent with RSWA. During one of the dream-enactment episodes that was recorded during REM, the patient appeared to self-soothe by stroking her head (Supplementary Video). Upon awakening, she reported dreaming about being upset and then, of being comforted, demonstrating concordance between dream content and observed motor behavior.

Trials of pharmacological treatment included prolonged-release melatonin (2 mg nocte) and, subsequently, vortioxetine (15 mg once daily). Both led to significant reduction in dream-reality confusion, improved overall cognitive functioning, and reduced post-awakening confusion. However, symptoms of immersive and vivid dreaming remained. Structured neuropsychological assessment revealed no evidence of dissociation, psychosis, or cognitive impairment. Similarly, neurological evaluation did not show anything of note. Screening for psychotic symptoms was negative, with a score of 0 on the psychosis module of the Mini International Neuropsychiatric Interview (MINI), and no evidence of delusional ideation or hallucinations on clinical interview.

## 3 Discussion

Presented is a distinct REM parasomnia case characterized by young-adult onset RSWA with RBD, dream enactment, persistent dream-reality confusion, and immersive dream narrative (see Supplementary for further examples). The absence of trauma, affective illness, or neurodegenerative disease distinguishes this presentation from PTSD or other trauma-associated parasomnia and classical idiopathic RBD (iRBD; Husain et al., 2001). At the time of initial evaluation, conducted 7 years after symptom onset, there was no evidence of parkinsonism or cognitive impairment. Consistent with this, a case series of 12 young patients with iRBD, including five children and seven adolescents or young adults (ages 18–25), also found no clinical signs suggestive of neurodegenerative disease at the time of evaluation, despite the presence of complex and heterogeneous clinical profiles (Shukla et al., 2019). Together, these observations raise the possibility that early-onset RSWA/iRBD may comprise a clinically and biologically distinct entity, phenotypically similar to classical RBD, yet etiologically independent of the prodromal neurodegenerative pathway that characterizes late-onset forms.

Clinically, dream-reality confusion in this context carries significant functional consequences. While phenomenologically similar to narcolepsy and dissociative states, our patient did not exhibit hypnagogic hallucinations or cataplexy. Patient's immersive, emotionally neutral, and predominantly mundane, dreams also differ from the affect-laden mentation observed in nightmare disorder or trauma-related REM disturbances (Levin and Nielsen, 2007). Thus, rather than indicating emotional overload, these dreams may suggest an atypical persistence of internally generated REM mentation into waking cognition (Wamsley and Stickgold, 2010). Arguably, the clinical features observed in this case may be interpreted as consistent with a variant of epic dreaming, potentially overlapping with the atypical end of the RBD spectrum.

One possible mechanistic explanation could involve disruption in the post-REM processing of dream content. Under normal conditions, REM-associated dreams, despite their vividness, are commonly easily forgotten (Nir and Tononi, 2010). Convergent animal work indicates that a subset of melanin-concentrating hormone (MCH) neurons in lateral hypothalamus is selectively active during REM sleep and supports forgetting of hippocampus-dependent memories (Izawa et al., 2019; Jego et al., 2013; Hassani et al., 2009). Using fiber photometry in freely behaving mice, Izawa et al. (2019) showed REM-linked activation of MCH neurons; chemogenetic and optogenetic, state-specific inhibition during REM impaired forgetting without altering gross sleep architecture (Izawa et al., 2019). Conversely, activating MCH neurons promoted forgetting (Izawa et al., 2019). Translating cautiously, a relative failure of REM-active MCH signaling in humans could allow unusually persistent encoding of REM mentation, aligning with our patient's intrusive recall and dream–reality confusion (Izawa et al., 2019; Luppi et al., 2024).

**Figure 1:**
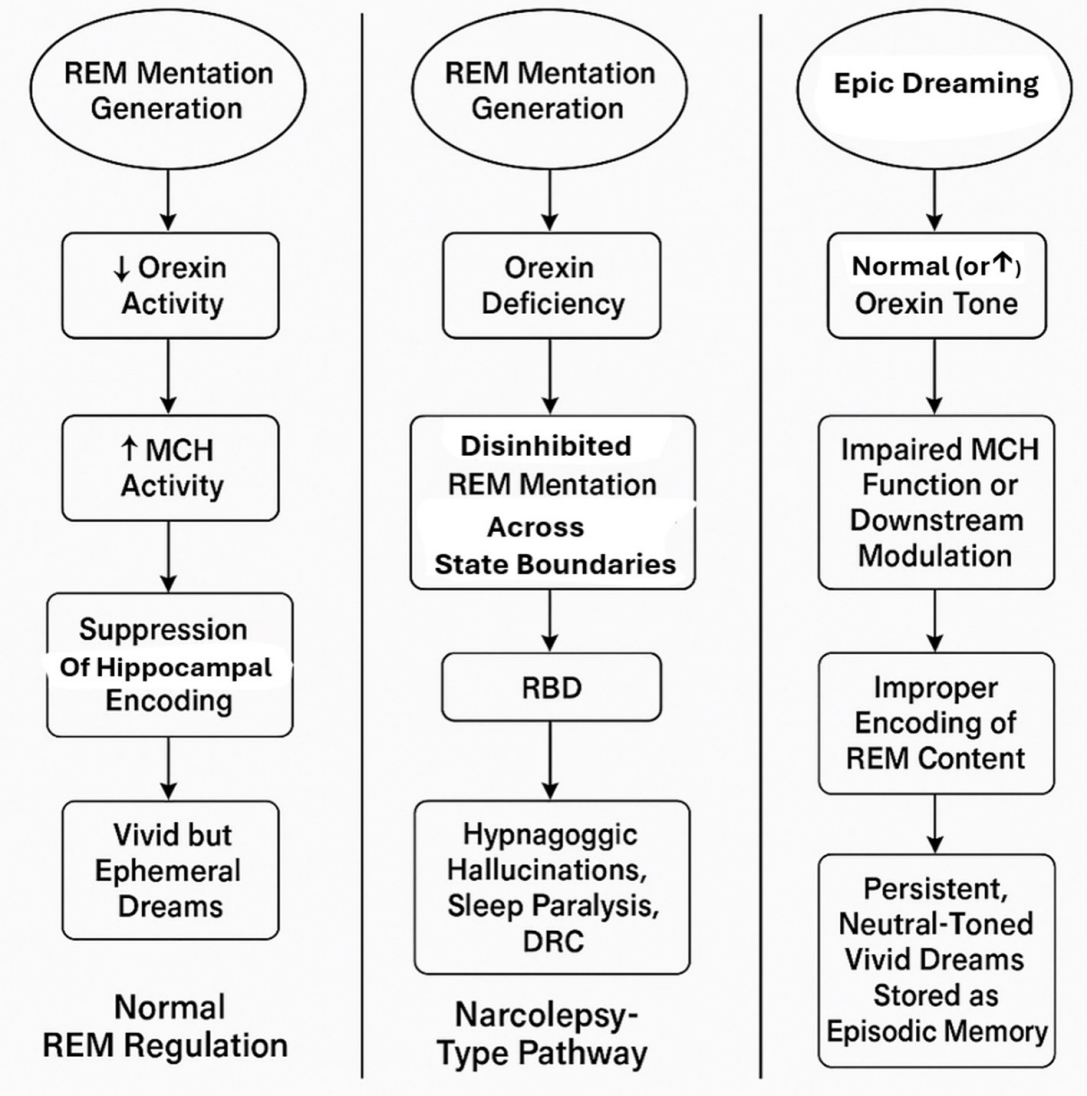
Integrated model of REM mentation regulation and pathways to dream-reality confusion (DRC). This schematic illustrates three hypothesized pathways linking REM mentation, orexin and melanin-concentrating hormone (MCH) signaling, hippocampal encoding, and the development of dream-reality confusion (DRC). ***(Left panel***) Normal REM regulation: reduced orexin activity during REM sleep enables MCH-mediated suppression of hippocampal encoding, resulting in vivid but typically ephemeral dreams. (***Middle panel***) Narcolepsy-type pathway: orexin deficiency permits disinhibited REM mentation across wake-sleep boundaries, contributing to REM sleep behavior disorder (RBD), hypnagogic hallucinations, sleep paralysis, and DRC. (***Right panel***) Epic dreaming phenotype (present case): despite preserved orexin tone, impaired MCH function or downstream modulation is hypothesized to allow abnormal encoding of REM content into episodic memory, leading to persistent, neutral-toned immersive dreams and pronounced dream-reality confusion. Moreover, theoretically, increased orexin tone, whether state-dependent (e.g., stress, sleep deprivation) or trait-related, could similarly lead to impaired MCH function.

So far any direct evidence for this mechanistic scaffold in humans is currently lacking (see Figure 1). In the present case, the suggestion of impaired dream forgetting remains a hypothesis that would require dedicated functional imaging or neurochemical studies to support. Nonetheless, this theoretical framework may offer a useful lens through which to interpret similar cases.

Past studies have highlighted the putative role of thalamocortical oscillations and sleep spindle dynamics in memory consolidation and dream recall (Steriade and Timofeev, 2003). Although spindle activity was not assessed in our patient, reduced spindle density has been linked to heightened dream recall and nightmare frequency in individuals with fragmented sleep, plausibly reducing ‘gating' of REM mentation into waking memory (Picard-Deland et al., 2018). We note this as a target for future longitudinal studies, alongside mechanistic hypotheses involving MCH–hippocampal pathways (Luppi et al., 2024). If present, such disruptions may facilitate the persistence of dream content into waking consciousness. Given our patient's childhood sensory vulnerabilities and evidence of fragmented sleep, a contribution of altered spindle regulation remains a plausible, although unconfirmed, element.

Partial improvement with vortioxetine in this case is potentially suggestive of serotonergic modulation of REM-related phenomena. Vortioxetine's pharmacological profile includes serotonergic reuptake inhibition and receptor modulation, which may influence prefrontal and limbic circuits involved in emotional contextualization and memory gating (Sanchez et al., 2015). However, while it is unclear whether vortioxetine exerts direct effects on REM circuitry, its partial effect may also reflect enhanced executive control or cognitive flexibility, rather than a direct modulation of RSWA or dream processing mechanisms (McIntyre et al., 2014). Classically, serotonergic reuptake blockade increases synaptic 5-HT and suppresses REM sleep (longer REM latency, reduced REM percentage) across patients and healthy volunteers (Wichniak et al., 2017). In healthy men, vortioxetine (20–40 mg) delayed REM onset and reduced REM time in a dose-dependent manner, with a profile distinct from paroxetine, likely reflecting its multimodal actions (e.g., 5-HT_3 antagonism; Wilson et al., 2015). Recent adolescent vPSG data similarly report increased REM latency and decreased REM percentage after vortioxetine treatment (Mlyncekova et al., 2023). Beyond macro-architecture, serotonergic input can facilitate spinal motoneuronal excitability via 5-HT-dependent mechanisms, which could, in principle, interact with REM atonia circuits (Perrier and Cotel, 2015). Importantly, several studies associate serotonergic antidepressants with increased RSWA in some patients; thus any anti-confusional benefit from vortioxetine should be interpreted as primarily cognitive/affective unless serial PSG demonstrates normalization of REM motor atonia (McCarter et al., 2015; Lee et al., 2016) In our case, vortioxetine improved dream–reality confusion whereas immersive dreaming persisted, aligning with a cognitive rather than purely motor mechanism. We did not quantify sleep spindles or repeat PSG following vortioxetine initiation; future longitudinal assessments will examine spindle metrics and RSWA indices in parallel with symptom change. Importantly, we here advance that even if distinct mechanisms underlying this specific phenotype remain unclear, they align with emerging views that RBD-like phenomena may arise not solely from neurodegeneration but also from more subtle neurochemical imbalances (Jiménez-Jiménez et al., 2021).

More broadly, this case emphasizes that RBD as a REM parasomnia can present with disabling cognitive and affective features even in the absence of violent or injurious behavior, and possibly, without any association to narcolepsy, nor to any other neurological disorders, including a neurodegenerative process such as alpha-synucleinopathy. However, long-term follow-up would be needed to fully address this issue, as neurodegenerative disorders often evolve slowly over decades, with the cardinal sleep manifestations, e.g., RBD and its spectrum disorders, emerging early in the clinical course. Current diagnostic systems, such as the ICSD-3-TR (AAoS Medicine, 2023), emphasize motor manifestations while neglecting metacognitive disturbances like dream-reality confusion (Avidan and Kaplish, 2010). Expanding diagnostic criteria to reflect the cognitive and affective dimensions of REM sleep dysregulation may allow for earlier recognition and more nuanced treatment strategies. Encouraging the systematic reporting and phenotypic characterization of similar cases will be essential to inform and support future revisions of diagnostic frameworks.

From a therapeutic standpoint, effective interventions for such presentations remain elusive. Neither melatonin nor serotonergic antidepressants yielded full resolution in our patient. Cognitive-behavioral strategies targeting reality monitoring may offer promise but require empirical validation. Future therapies might aim to modulate REM memory encoding directly, pending advances in our understanding of these processes.

In conclusion, we describe an idiopathic and non-neurodegenerative case of RBD, with features of epic dreaming, and with marked cognitive and affective symptoms not currently captured by existing nosologies. While supported by objective evidence of RSWA, the hallmark features lie in the persistence, content, and behavioral impact of REM sleep mentation. This case underscores the need for refined clinical frameworks and targeted research into the cognitive consequences of REM dysregulation.

## Data Availability

The original contributions presented in the study are included in the article/Supplementary material, further inquiries can be directed to the corresponding author.

## Supplementary material

Supplementary Table S1 Phenomenological Matrix.

Supplementary Video S1 Dream enactment behaviour in a patient with dream-reality confusion. This segment of video-polysomnography (vPSG) illustrates a brief episode, where patient performs repetitive movements with the right arm (touching her hair and forehead), then lifts her left arm and finally moves her legs (first she adducts her legs and then extends them, and finally rotates the pelvis and the lower limbs to the right). The episode lasted approximately 8 seconds. At EEG level the ongoing activity is completely masked by motion artifacts. The ECG trace documented a mild acceleration of heart rate during the hypermotor manifestation. The montage includes the following channels, displayed from top to bottom: electrooculography (EOG; E1–M1 and E2–M2), electroencephalography (EEG; F3–M2, F4–M1, C3–M2, C4–M1, O1–M2, O2–M1), electrocardiography (ECG-LA–ECG-RA), submental electromyography (EMG; CHIN1–CHINz, CHIN2–CHINz, CHIN1–CHIN2), bilateral anterior tibialis EMG (RLEG+-RLEG, LLEG+-LLEG), airflow (XFlow), snore and flow digital recordings (Snore_DR and Flow_DR), thoracic and abdominal effort plethysmography (Chest and Abdomen), pulse oximetry (SpO_2_, %), and pulse rate (PR, bpm). Notably, the submental and limb EMG channels reveal elevated and sustained muscle activity during REM sleep, consistent with REM sleep without atonia.
