# Supplementary material for: A non-neurodegenerative REM parasomnia with immersive dreaming and dream-reality confusion: a case report
Source: Front Sleep. 2025 Sep 11;4:1659300. doi: 10.3389/frsle.2025.1659300 (PMC12713830; doi:10.3389/frsle.2025.1659300)
Supplement: Supplementary Table S1 — Phenomenological Matrix. [file Table_1.docx]

Supplementary Material

To further illustrate the phenomenological structure and cognitive-affective burden of the patient’s immersive dreaming, a thematic analysis of their contemporaneous dream diaries is included as Supplementary Table S1. This matrix links core dream elements, such as spatial immersion, narrative layering, and dream-reality confusion, to established constructs in dream neuroscience, psychopathology, and cognitive-affective theory. All examples have been anonymised and restructured to protect identity while preserving their scientific integrity and phenomenological richness.

# Supplementary Table S1: Phenomenological Matrix

| **Dream Element** | **Description** | **Psychopathological Equivalent** | **Commentary** |
| --- | --- | --- | --- |
| **Spatial immersion** | Highly detailed dream environments that mimic real-world urban or domestic locations, including specific transport systems, offices, and furniture. | Autoscopic spatial misattribution; hyperassociative continuity | Supports the hypothesis that hippocampal-encoded spatial memory traces are active and possibly retained abnormally post-REM. |
| **Multilayer narrative** | Dreams contain nested layers (e.g., dreaming of recounting the dream), or reflexive narration of ongoing dream activity. | Nested hallucinosis; oneiric metacognitive layering | Suggests altered metacognitive gating and lucidity-like cognitive operations in immersive dreams without full awareness. |
| **Dream-to-wake influence** | Waking from a dream state with lingering cognitive-emotional states or sensory impressions that feel indistinguishable from waking memories. | Pseudo-episodic memory formation; hallucinoid persistence | Reinforces the concept of dream-based 'false memories' with strong affective realism but intact insight post-waking. |
| **Reality-testing** | The dreamer engages in checks (e.g., emails, text logs) to confirm if dream events actually occurred. | Compensatory insight behaviors; non-psychotic confabulation | Illustrates a non-pathological form of reality monitoring failure, possibly mediated by insufficient dream erasure mechanisms. |
| **Dream impostor syndrome** | The dreamer experiences work-related and interpersonal anxieties embedded in dreams with themes of surveillance, judgment, or evaluation. | Self-referential misattribution; delusional mood mimicry | Constitutes a realistic, non-delusional analogue of social evaluative threat encoded during REM mentation. |
| **Autonoetic disorientation** | Difficulty determining agency and self-boundary in dreams, such as when dreams replay or mimic waking behavior without subjective awareness. | Dereality threshold breach; ego-boundary permeability | Reflects a borderline dissociative structure, but without fragmentation of consciousness or psychotic symptoms. |

# Dream Vignette

‘*Really long dream that I remember snippets of- walking to the station in the cold / slightly snowy environment. Except I was walking to a different station from normal and had to walk past one and keep going to the next. The first one had trains that only went to XX and I needed the XY train (in reality the AB train). I was walking on the side for trains out of AX though. Not a station I recognised. On the platform of that first station I walked past a work acquaintance whose name I can’t recall right now - ginger, braces, has a kid, works in compliance change.*

*Then the platform sort of continued so that you could keep walking to the next station. There was someone jostling a bit to overtake me, which made me nervous that I was running late for my train. I’d never taken the train from these stations before. I passed a coffee shack with someone sitting there. Looked like a converted countryside bus shelter (wooden 3 sided construction). There were various posters including one of train times, but I didn’t have time to stop and double check times.*

*The path to the next station stretched out ahead of me - snowy, meandering slightly uphill. There were other people ahead of me walking to the station so I continued.*

*Got to the next station. It was much busier and had multiple platforms. I was now on the right side for a journey to AB but there was a sense I was in Australia (?). Still didn’t recognise anything. The platform also looked weird. I straightaway locked eyes with someone and either struck up a conversation with a stranger or he was a slight acquaintance. He was in a motorised wheelchair on the edge of a platform and I was glad to stop and talk to him because I had an impression that if I was hanging out with him then we’d get on the train on a sort of pre-platform that it stopped at for disabled people. And id probably then get a seat.*

*I was chatting with the disabled man and I think someone else who was there, not disabled. I’ve got a sense of one of them being XX from work, a distant colleague who I like and respect. In the chat with him / them it included that when I’d been skiing last I’d spent a few days skiing with a disabled person (totally not true).*

*A train approached that wasn’t for us and the wheelchair person got very agitated that the train driver had it in for him (XX? I don’t know a XY) and was going to purposely travel too fast and inaccurately and hit him. So as that train came through the disabled man leaned really far back in his chair sort of like it was on a spring and was swearing. We both / all looked at the back of the train as it disappeared.*

*Eventually I was on the train I think. General sense that I’m going to be late. Walked past some sort of long and strange coffee kiosk that was very weirdly set up. I thought a customer was there by the coffee but it seemed to be an employee saying to his boss quite rudely that the password for the WiFi had changed and why hadn’t he been told and sort of making up a reason for why he needed it, but it was clear that the reason was because he was a slacker. His boss was shouting at him on the phone including saying that he’d sent him an email with it.*

*I kept walking.*

*In the office now, not my actual office. Snippets.*

*We moved to a different row. Colleagues agreed it was better than the old seats.*

*Floor is being reconfigured. Looks more like a department store floor than work. I can see my colleague AB who looks after property picking her way through a section that’s been destroyed a bit - on the other side from me. She doesn’t see me. She’s picking up a few bits and bobs and putting them in a container that she then chucks down. She’s got slightly platform heeled chunky shoes on and there’s something stuck to the heel of one shoe. She turns away from me and heads off around a pillar out of sight.*

*I’m walking to the station in the evening. I pass my colleague AA talking to someone senior outside some sort of cafe. We aren’t in the city, more suburban. AA is saying that he hasn’t eaten as much today because he’s not done much exercise, though he is hungry. The senior person (who in the dream I feel I know, but now don’t think was an actual person I know) agrees with this approach and says everyone is always hungry. I feel embarrassed that I eat until satiated. Keep walking to the station.*

*It has been a long day. I’m ready to go home and a bit anxious about the journey. It’s getting dark again so I’ve been at this since early trying to get to work. It doesn’t seem snowy where I am now. But the coming twilight does appear related to the season, so it’s darker earlier.*

*I woke up tired and then went back to sleep, and dreamed I was telling you about the dream*.’
